# Reciprocity on Demand: Sharing and Exchanging Food in Northwestern Namibia

**DOI:** 10.1007/s12110-015-9236-5

**Published:** 2015-07-31

**Authors:** Michael Schnegg

**Affiliations:** Institute of Social and Cultural Anthropology, Universität Hamburg, Edmund-Siemers-Allee 1, D-20146 Hamburg, Germany

**Keywords:** Food sharing, Exchange, Social networks, Reciprocity, Africa

## Abstract

Two competing models concerning food transfers prominent in the anthropological literature conceptualize such transfers either as sharing or as exchange. Sharing is understood as situational transactions formed through demands and unconditional giving, whereas reciprocal exchange is understood in terms of networking and keeping score. I propose that the picture is more complicated than these classifications suggests. Drawing on data collected in Northwestern Namibia, I show that sharing and reciprocal exchange are dynamically interrelated in actual food transfers. As a local norm, people can demand food from anyone, and they are typically given food in response to a demand. However, in practice, food transfer networks emerge (*N* = 62) that are highly reciprocal and fit the exchange model much better. Although the sharing norm makes no restrictions on whom to ask, in practice people often turn to their neighbors. Interpersonal dynamics account for why some of those ties become strongly reciprocal and others do not. Under these circumstances, unconditional sharing, a norm that has been viewed as an alternative to exchange, can lead to reciprocity via reciprocity on demand.

We slaughtered the goat in James’s front yard and a neighbor passed by, calling, “Give me meat, James, give me some!” Without discussion, James put part of the innards into a plastic bag and handed them over. After the neighbor left, I asked James whether he would give some to anyone who demand in that way. James avoided a direct answer and replied: “You know, neighbors are important; we depend on one another.” This ethnographic vignette describes a food transfer in Fransfontein, Namibia.^1^ At first the blunt demand for meat seems to be in stark contrast to the exchange model that has long dominated anthropological understandings of food transfers. For example, Sahlins (1965:145) argued that food transactions, especially those after a successful hunt, are best described as generous exchanges in which the lucky “haves” give the “have-nots” in expectation of future reciprocation (Winterhalder 1986; Woodburn 1998:48). This interpretation contains two central components—generosity and reciprocity—and views transactions as strategic acts to buffer risk and reduce insecurity (Cashdan 1985, 1990; Wiessner 1982, 2002).

In recent decades, new data similar to that of the case of James and his neighbor in Fransfontein along with a critical reading of ethnographic literature have led many to question both assumptions. On the one hand, Peterson (1993), Bird-David (1990, 1992, 2005) and Blurton Jones (1984, 1987), building on Parker and Smith (Parker 1974; Smith and Parker 1976; Smith 1974), have provided evidence that food transactions are often solicited by a recipient, rather than generously initiated by the possessor. To account for this, they introduced the terms “demand sharing” (Peterson) and “tolerated theft/scrounging” (Blurton Jones) to make it clear that many transfers are explicitly demanded by a recipient. On the other hand, and on similar grounds, Woodburn challenged the second assumption, reciprocity. If a hunter has relatively little control over who gets what, then “giving” today does not create entitlement for receiving tomorrow (Woodburn 1998:57).

To mark this difference between strategically crafted exchange relationships and situational distribution to all who make demands on the giver, anthropologists have developed a sharing model that is complementary to and in some cases replaces the model of reciprocal exchange (Bird-David 2005; Widlok 2004, 2013; Woodburn 1998). As Woodburn has expressed it so clearly, sharing is not a form of exchange (Woodburn 1998). This debate, originating in hunter-gatherer studies around the transfer of food from large carcasses, has since reached out into other domains of food transfer. These include transfers of other foods, whether hunted or gathered, owned as livestock, or purchased in shops, and even include the transfer of non-food items. In these other domains, similar logics are observed (Bird-David 1990; Peterson 1993; Widlok 2004, 2013).

The dichotomy between sharing and exchange emerged in sociocultural anthropology, but most research in human behavioral ecology does not draw the same distinction. Here, “food sharing” typically refers to food transfers in general. Kin selection, reciprocal altruism, risk minimization, reputation, and tolerated theft have been proposed as partly competing and partly complementary models to account for the observed transfers (Gurven 2004; Nolin 2010; Ziker and Schnegg 2005). At the same time, the strain between reciprocity and the sharing of a “public good” was also debated (Hawkes 1993). In this article, I argue that it is analytically useful to depart from Woodburn’s terminological distinction and use “sharing” to refer to a specific type of distribution complementary to reciprocal exchange.

To return to the opening vignette, James’s reply to my question already indicates that the distinction between sharing and exchange is much more blurred than the anthropological literature suggests. James refers to a context, the neighborhood, as a factor that makes transactions with some more likely than with others. In addition, he explains that neighbors are important and depend on one another. Taken together, his answer reveals that durable and possibly reciprocal ties to specific others do exist even as demands for unconditional sharing are present. This example is consistent with many recent studies that have also reported high levels of reciprocity in comparable situations (Allen-Arave et al. 2008; Gurven 2004; Gurven et al. 2000; Koster 2011; Nolin 2010; Wiessner 2005:136; Ziker 2007).

In this article, I propose distinguishing between two levels, one normative and one practical, to explore whether and how sharing and exchange may be integrated. The normative level refers to rules and expectations for transactions in which food is distributed; the practical level starts with the actual transfers and describes the social networks that emerge from their reciprocation. But I also show that norms and networks are intertwined. A tit-for-tat norm should lead to a reciprocal network structure. In contrast, giving to all who demand situationally is more likely to result in networks that correspond to a random graph (Schnegg 2006).

The two levels—norms and networks—require different data for analysis. Norms can be reconstructed ethnographically through observations and interviews. Much of the literature builds on such evidence (Bird-David 2005; Marshall 1976; Widlok 2013). By contrast, actual practices can be described more precisely with quantitative data (Kaplan and Hill 1985). A promising way for doing so is through social network analysis (Allen-Arave et al. 2008; Koster 2011; Nolin 2010; Wood and Marlowe 2013; Ziker and Schnegg 2005). Social network analysis is a growing interdisciplinary paradigm that provides analytical tools for describing and modeling social structure (Schweizer and White 1998; Wasserman and Faust 1994). A network N = (V,E) is defined as a set of vertices (V) and a set (E) of unordered pairs of distinct elements of V called *links.* In the case of food sharing, links are food transactions and the vertices are individuals or households.

As the opening vignette indicates, transacting food is a daily routine for people in and around Fransfontein, Namibia. On average, every household engages in one or two transfers a day.^2^ The foods transacted are often basic goods—most importantly, sugar. As in many poverty-ridden environments, sugar is an important source of caloric intake, and many people depend on it for daily subsistence (Mintz 1985; Scheper-Hughes 1992). The people in Fransfontein refer to these exchanges as *augu*. The word *au* derives from the Afrikaans word *ou*, which means “to give.” As with a number of Afrikaans and German words, it was introduced in Khoekhoegowab, the indigenous language, during colonial rule. Today, the usage in Khoekhoegowab entails the assumption that a transaction is commonly initiated by the one who wants and is therefore the one likely to receive. The suffix -*gu* is referred to as reciprocal by linguists and signifies that the relationship goes both ways. It is commonly used by Khoekhoegowab speakers to indicate than an action (verb) is reciprocal: for example, “to teach” (*||khā||khā*) becomes “to teach each other” (*||khā||khā-gu*) through the suffix *-gu*.

Against the background of the debate sketched above, my aim in this article is to explore *augu* as a way of sharing and/or exchanging food.^3^ After a brief introduction to the ethnographic setting and the research design, I describe the normative order ethnographically. Next, I introduce a set of network measures to analyze the social structure that emerges from *augu* practices. As the analysis reveals, norms hold that one can demand from anyone else and that one should share with everyone as well. At the same time, the networks that emerge do not match the sharing norm. In contrast, they are highly structured, and most transactions are embedded in reciprocal ties. Those patterns can be explained if one takes the social context and the temporal dynamics of relations into account. These factors help in understanding how and why *augu* is a form of reciprocity on demand.

## Ethnographic Context

Fransfontein is a community of roughly 150 households in Northwestern Namibia extending over about 10 ha. The communal pastures surrounding it are dotted with small settlements of 3 to 19 homesteads. They cluster around drilled boreholes that provide water for humans and their livestock in the semi-arid environment (Schnegg and Linke 2015; Schnegg et al. 2013). Most inhabitants of the larger Fransfontein area consider themselves as Damara or Nama people (Dawids et al. 2007). Before contact with the German colonizers in the late nineteenth century, the Damara were most likely hunter-gatherers, with significant contributions to their economy coming from small-scale trading. Their main diet consisted of plants, insects, and large game, and people still have significant ecological knowledge in those domains (Lau 1987; Sullivan 2004). The ||Khau|gôan (Swartboois) Nama arrived in the area around 1880. At that time, most Nama combined pastoralism with trading and raiding (Lau 1987). Under the supervision of German missionaries, the ||Khau|gôan started gardening and established the infrastructure of the community (e.g., church, school, shops) as it is today. Both groups share the same language, Khoekhoegowab (Barnard 1992:11). For the area around Fransfontein, intermarriages and migration have established a society in which many cultural models and practices are shared. This is also true for food sharing, and we were not able to identify differences between ethnic groups (Dawids et al. 2007:27). Unfortunately, I cannot say whether practices differed in the past and how they possibly converged. Consequently, the ethnographic analysis does not distinguish between ethnic groups.

In Fransfontein only a small elite, mostly teachers and public servants, can count on a steady income. In addition, all people age 60 and older received a minimum pension payment of 250 N$ from the state at the time the quantitative data were collected in 2004.^4^ Most households depend for their livelihood on a combination of different social and economic strategies. Many households combine pastoralism, wage labor on commercial farms, migration to urban centers, and state transfers to reduce economic risks and vulnerability (Greiner 2011). However, even those flexible and adaptive strategies do not prevent the majority of households from living in precarious conditions (Schnegg et al. 2013).

Fransfontein has a handful of small stores where basic food items, including sugar, tea, and maize meal, can be purchased. However, the shops are seldom used because most households have cash on hand only a few days a month. Even those who receive regular pension payments, wages or salaries spend their money the first day or two after receiving it. When people receive their payments, they typically buy the most basic staples first: maize meal, tea, and sugar, in quantities that are expected to last for the month. For a household of five a typical first shopping trip would include 20 kg of maize, 5 kg of sugar, and a box of tea. Although enough maize meal can typically be stocked for the month, sugar and other more-valued items (e.g., tea, coffee, tobacco) are more problematic. Sugar is much sought after, and if plenty is kept in the house, the level of consumption rises. After these basic supplies are purchased, the rest of the money is used to buy other goods, such as tobacco, soap, and deodorant. In addition, fees for school are paid, the health clinic may be visited, and other important activities may be accomplished as financial resources allow.

Payday is also the time when debts that have accumulated during the month are paid off. Some shops and private businessmen and businesswomen sell goods on credit during the remainder of the month and demand their money as soon as they know it is available. Credit is given without charging interest, but only to those who have the reputation of paying off their debts. In addition to such private debts, those who are better off, such as teachers and civil servants, usually have loans with furniture stores (James 2014). On payday the atmosphere in Fransfontein turns festive and the local bars are well frequented. However, this spending commonly lasts only a short while as money reaches the cashboxes of those few men and women who run businesses in Fransfontein. There it stays and is rarely reinvested locally. For the rest of the month food transactions become important and constitute a significant social strategy.

## Data and Network Measures

Most of the qualitative and quantitative data presented here were collected during 18 months of ethnographic fieldwork between 2003 and 2006. During that time, my wife and I and my colleague, Julia Pauli, lived in Fransfontein. As members of the community we were able to observe and participate in many of the practices described here on a daily basis. I returned to Fransfontein for shorter stays, especially after 2010 when I started a comparative research project with my colleagues on water and land management in Northwestern Namibia. Some of the interview data presented here were collected during those more recent stays.

I apply social network analysis to capture the flow of transfers between households. In 2004 over a period of 10 days our research team (consisting of myself, Julia Pauli, Francois Dawids, Valery Somses, and Jorries Seibeb) visited 43 households in Fransfontein and 19 in two farming communities in the surrounding hinterland. The households in Fransfontein were chosen on the basis of almost 1 year of ethnographic work with the aim of representing different economic strategies and strata. I thus follow an ethnographic sampling approach that aims to capture variations and heterogeneity in the population (Werner and Bernard 1994). For Pos 66 (13 households) and Olifantwater (6 household), two communal settlements surrounding Fransfontein, all households were interviewed. Every morning someone from our team visited each of the 62 households and questioned mostly the female head of household about their transactions during the previous 24 h. Those transactions might include engagements with households in our sample or with others who are not part of the sample. The data base consists of 1,631 transactions. When we eliminate demands that could not be met, the number drops to 1,487. During the interview we did not restrict data collection to transactions that were classified as *augu;* we included *len* as well. *Len* refers to borrowing a good, mostly tools and equipment, that is expected to be returned. The following analysis is based on 1,087 transactions classified as *au*.

The data presented here differ in some ways from much of the data on food sharing discussed in the literature. Many previous studies have relied on participant observation (Bird-David 1990; Widlok 2013) and/or the analysis of dyadic relationships (Gurven et al. 2000; Kaplan and Hill 1985). While participant observation is crucial for understanding transactions, it does not allow for quantification of the exact flow of goods. The analysis of dyadic relations, in contrast, does. These data allow researchers to address such questions as the average degree of genetic kinship between two people who give or receive food and whether the quantity of food being transacted increases with the closeness of kin ties. However, although these analyses are sophisticated at the dyadic household-to-household or person-to-person level, they lack information about absent ties: those who do not transact food. Thus, it is difficult to reconstruct a network structure from the data (Allen-Arave et al. 2008; Koster 2011; Nolin 2010; Wood and Marlowe 2013; Ziker and Schnegg 2005), which is crucial to understanding some of the properties of a system and to distinguishing between sharing and exchanging.

Table 1 lists three different measures used to describe these networks and to distinguish between sharing and reciprocal exchanges as they have been presented in the anthropological literature. The level of reciprocity provides a first indicator to test which of the two models match the data observed. I use two different approaches to measure reciprocity. The descriptive measure computes the number of reciprocated and unreciprocated ties and determines their proportion (Pryor and Graburn 1980; Schweizer 1997; Ziker and Schnegg 2005). However, even if a number of people in a group interact randomly, some level of reciprocity will still occur, depending on group size and the number of transactions and the distribution of in- and outgoing flows (Blurton Jones 1987:38; Schnegg 2006; Wasserman and Faust 1994:547). When the number of transactions rise, the likelihood of reciprocation by chance rises as well. The second and more sophisticated measure therefore estimates the level of reciprocity that would be expected by chance in a network of a given density and distribution of in- and outgoing ties and compares it with the observed.

**Table 1:** Qualities distinguishing sharing from reciprocal exchange as these modes of food transaction are defined in the anthropological literature

Indicators	Sharing	Reciprocal exchange
Reciprocity: descriptive	low	high
Reciprocity: modeled	less than expected by chance	more than expected by chance
Density	high	low

A third indicator that distinguishes sharing from exchange in the literature is a network’s density. Density is defined as the number of present ties in proportion to the number of possible ties. The number of possible ties depends on the size of the network and equals *N*(*N−*1), where *N* is the size of the network. If ties are reciprocated and the reciprocity concentrates in a few relationships (exchanges), we would expect relatively low densities. Density can thus serve as a third indicator to distinguish sharing from exchanging. Before we can use these indicators to determine which model best describes *augu,* I deal with the norms that govern *augu* transactions.

## Results

### Norms

Table 2 gives an overview of the distribution of goods transacted among the 62 households during a 10-day period in March. Some of the *augu* transactions include non-food items. I did not exclude them from the analysis because the same cultural logics apply.

**Table 2:** Distribution of goods being transacted over a period of 10 days (*N* = 1,087)

Items	*N*	%	Cumulative %
Milk	125	11.9	11.9
Sugar	124	11.8	23.7
Coffee/Tea	109	10.4	34.1
Tobacco	93	8.8	42.9
Firewood	81	7.7	50.6
Meat	75	7.1	57.8
Maize	52	4.9	62.7
Spices	41	3.9	66.6
Bread	35	3.3	69.9
Vegetables	33	3.1	73.1
Money	32	3.0	76.1
Salt	31	2.9	79.1
Snuff	30	2.9	81.9
Oil/Fat	28	2.7	84.6
Medicine	26	2.5	87.1
Tools/Repair	26	2.5	89.5
Other food item	23	2.2	91.7
Others	123		100.0
Total	1087	100	

Since the goods transacted could not be weighed, it is difficult to quantify them. Fortunately, for many goods an informal standardization exists that helps to grasp the amount and the context of these transfers. Number one in the list is milk because the data were collected during the rainy season, when cattle usually have calves. At that time milk is relatively abundant (and fat), which makes it a much-desired good. In contrast, during the rest of the year cows are rarely milked. Milk is mostly transferred from the more rural cattle posts to Fransfontein. Common measures and modes of transport are 1- or 2-l Coca-Cola bottles. The sour milk is mixed into cooked maize meal (porridge) to add both flavor and fat.

Sugar, number two on the list, is almost always exchanged in drinking cups that hold 200 ml. In Fransfontein, as in many comparable environments, sugar means much more than the luxury of sweetness (Mintz 1985). For most people it is an important calorie intake. Most people start the day with a cup of hot water or tea mixed with two to three tablespoons of sugar. One cup of sugar (roughly 800 kcal) covers the daily need of a household of five people. The two goods that follow in Table 2 are tea and two nonfood items, firewood and tobacco. In general, the table reveals that most of the goods transferred satisfy basic needs.

The transactions for two of the goods listed in Table 2 are restricted to daylight hours. Firewood and salt cannot be exchanged after dusk; people are convinced that good luck (*!gâi!gâb*) would leave the house along with the good. This rule is embedded in a more general understanding that *!gâi!gâb* can be lost at night. For example, sweeping dust and sand out the door after dark is also perceived as dangerous. It is difficult to say why the understanding is restricted to these few items. Both were important and scarce goods in precolonial times. People explain that in the past it was not the wood but the fire itself that should not be given away after dark. When people left the homestead during the day for hunting or gathering they kept their fire covered with sand to avoid its going out. Salt had to be imported from the coast, a lengthy and dangerous trip through the Namibia desert, which made it quite scarce.

As I outlined above, *augu* transactions take place in different social contexts. The situations are generally characterized by the fact that some good (in the opening example it is meat) is unequally distributed in a given situation. One actor has this good in relative abundance and the other demands a fair share. Most of the transactions of milk follow this cultural logic. How many cows a household owns is common knowledge, and wealthy cattle owners are often confronted with demands to share some of their milk. Those who demand are typically visitors to Fransfontein from rural settlements, where few cows are kept and milk is scarce. When milk is plentiful, a demand is seldom denied.

This applies to other situations as well. For example, Mopani worms (*Gonimbrasia belina*) are abundant in some areas around Fransfontein at the end of the rainy season. Being tasty and fat, they are valued as a delicacy. Similarly, game meat is commonly available as a result of tourist activities on commercial hunting farms or illegal hunting. At the same time, for those not working on a hunting farm or engaging in poaching, meat is a scarce good in the daily diet. People are reluctant to slaughter their animals for consumption other than during festivities. In all three cases a good that is known to be plentiful in a certain context becomes subject to demands. It is difficult to withhold such goods from those who do not have them.

Throughout the year, the most common *augu* transaction occurs when the morning tea or other meals are prepared. That is when sugar is needed. The person who is preparing the meal, usually an older woman, is likely to send one of the younger children of the household to another homestead to issue a demand for a missing ingredient, such as sugar. The child will run over to the other household, often carrying a container, and say “*Au te re sukur-e*” (Give me some sugar). S/he will neither say “please” nor “thank you.” This indicates that the person has a right to demand and also that the property relations and the character of the good are in some sense communal.

Another common *augu* transaction occurs when food is being prepared outside. When porridge is being cooked, only a single pot is needed. But when people notice a household using two or more pots, meat or some other food is being prepared as well. A person walking by may just sit at the house or near the pot, talking to the cook and waiting until the meal is ready to be served. At that time, it is impossible to deny a share.

As in other comparable situations, hiding food or other goods is a common strategy to avoid sharing (Bird-David 1990:191; Durham 1995:125; Holmberg 1969:87; Levi 1999:93). The existence of such strategies indicates how strong the expectation to distribute food is. In Fransfontein, the practice of hiding applies not only to hunted or gathered food but to groceries bought in stores as well. Fransfontein has a small number of shops where the most basic items can be purchased. Even in those shops that operate out of private houses built with sticks from mopani trees (*Colophospermum mopane*), mud, and cow dung, all groceries, even a pack of tea, are wrapped in dark plastic sacks. The purpose is clear: to keep their purchases from being seen on the way home. When I asked Tia why she always buys in the shop next door even though the shop on the other side of Fransfontein offers much better prices, she replied that this way she does not have to walk through the entire community with her groceries. For similar reasons those who have a steady income as teachers or governmental employees prefer to shop in towns as far as 150 km away, where they are not observed in the supermarket. They also have locked cupboards and refrigerators in their kitchens to keep items (including soap, deodorant) out of sight (Austin-Broos 2003:126).

Many of these observations point to a transactional logic that Klocke-Daffa (2001) has summarized for the Nama of southern Namibia: “If you have, you must give.” Or as Tina, a 65-year-old pensioner and well respected church elder puts it: “But when we grew up, our parents, like my mother, who raised me, they said: ‘if you have, you must give it to someone who comes to ask for it.’”^5^ People place no restrictions on this value. They most often replied to the question “To whom must one give food?” with the quip, “To everyone.”

Giving food to others is often conceived of in terms of need. For example:

MS: If you do not give, although you have something at the house, how does that feel?

Erika: I will feel guilty, because I have, and that person ask for it, but maybe I said “No,” [and I] didn’t give. Then afterwards I will feel guilty: “Why didn’t I give [to] him? Because I have this, and he is in need. Maybe the children at home are hungry,” you feel guilty.^6^

Erika puts giving food in the context of suffering and hunger. Almost all people in Fransfontein have experienced hunger over extended periods of time. This then is a shared experience. To give to someone who is in need is not only a socially enforced norm (Wiessner 2005) but also internalized through shared experiences of suffering.

Equally, people can request from anyone. For example, when I conducted interviews in more remote areas my assistant Jorries typically accompanied me. When we approached households in the rural hinterlands we could often easily see whether meat, an occasional and much-sought-after variant in the daily diet, was available. If two pots were in use at the hearth (as noted above), Jorries would ask whether the dogs had chased a kudu (*Tragelaphus strepsiceros*, a reference to poaching, which is carried out with dogs) or whether the household had slaughtered a goat. Once the suspicion was confirmed, he readily demanded a fair share, independent of his relation to the household. In the course of fieldwork I learned that the same was expected of me, and I began asking for meat as well.

In a nutshell, the analysis of this ethnographic material enables me to assess the normative dimension of food transactions in Fransfontein quite clearly. Almost all transactions begin with a demand. And, in most cases, the demand leads to a transfer. In that sense, *augu* seems a perfect case of what Peterson (1993) has introduced as “demand sharing.” Quantitative data allows further testing of how these norms map out in the networks produced.

### Networks

I have shown that the level of reciprocity can distinguish sharing from exchange. If sharing were the dominant model governing transactions, one would expect a relatively low level of reciprocity. In contrast, if exchanges were dominant, one would expect reciprocity to prevail. To measure the level of reciprocity among transacting units I concentrate on the inner core of the network containing the relationships among the 62 households interviewed. From the 1,087 transactions observed, 508 (46.7%) took place among the 62 households interviewed. Those transactions form 180 relationships. In the dyad-based method, the reciprocity value indicates the proportion of dyads that are reciprocal, i.e., Num(X_ij_>0 and X_ji_>0)/Num(X_ij_>0 or X_ji_>0). In the *augu* network 44.0% of the ties are reciprocated. The level is higher than in any of the six other cases previously reported (Schnegg 2006).

A more sophisticated test of reciprocity compares this observation with the level of reciprocity expected by chance. In the first place, a test for reciprocity is carried out by testing the number of mutual dyads, M, in the U | X_i+_, X_+i_ distribution. Each network can be divided into combinations of three, called “triads.” Analytically one can identify 16 different triads, and 9 of those are mutual: that is, they contain a reciprocated tie (Wasserman and Faust 1994:566). For the *augu* network, the observed value of mutual triads is 55. Still, this number does not reveal whether or not one would expect a similar level of reciprocity by chance. To test the significance of such a finding Snijders has proposed a procedure that simulates matrices of equal size conditioning the distribution of in- and outgoing ties of each node (Snijders 1991). This procedure (ZO) is implemented using the StOCNET software package. The results after 10,000 simulation runs show that on average 6.87 mutual triads have been observed in the simulated matrices. The estimated variation is 5.67 and the estimated standard deviation is 2.38. In none of the 10,000 simulation runs is reciprocity as high as the value observed in the data. These results thus clearly confirm that *augu* transactions are about eight times as reciprocal as expected by chance!

I have introduced network density as a third indicator distinguishing sharing from exchange. Recall that the inner core of the network contains 508 transactions. If they never overlap, one would expect the density of the dichotomized network to be 508/62*61 = 0.13. The observed value, 0.05, is less than half of it and indicates again that the practices are better characterized as exchange than as sharing.

Table 3 compares models and results obtained from the network analysis. The first columns repeat Table 1; the third gives the empirical results and whether those values match with the model of sharing or exchange. It reveals that the network transactions exclusively match the notion of exchanges as defined in the literature and implemented here.

**Table 3:** Models and social network results compared

Social Network Indicators	Sharing	Exchange	*Augu* result	Fits Model
Reciprocity: descriptive	low	high	high	exchange
Reciprocity: modeled	low	high	high	exchange
Density	high	low	low	exchange

**Fig. 1:**
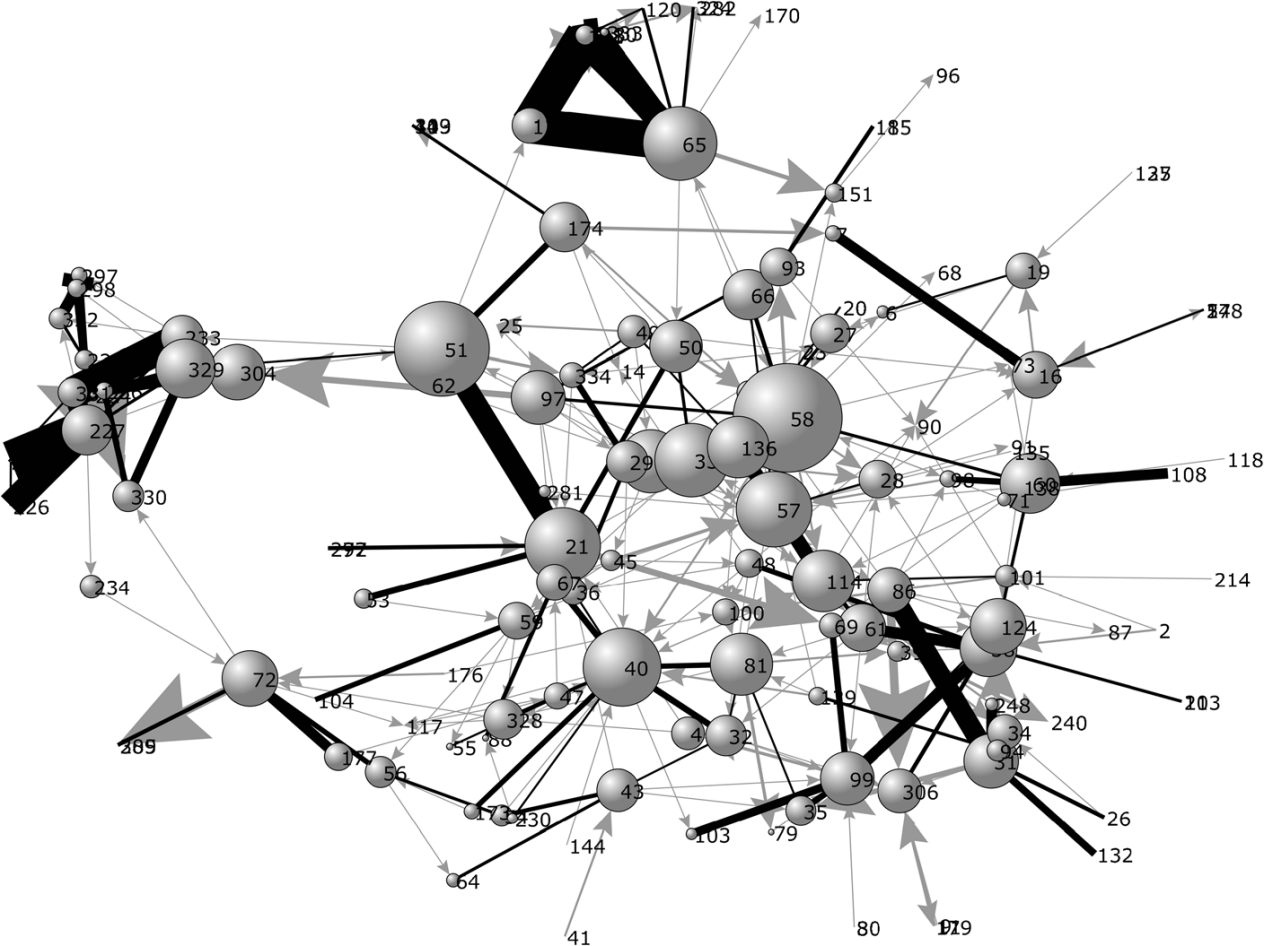
Network of food transactions (*N* = 143)

When the transactions the research team identified are graphed, they form a complex network (Fig. 1).^7^ The figure shows the households as nodes connected through transactions. The layout of the graph is not spatial. It uses a spring embedding algorithm to place nodes with multiple food transactions close to one another and those with few food transactions farther apart. The algorithm thus aims to detect the social structure of food transactions and produces a social map of it. The graph in Fig. 1 not only includes the relationships among the 62 households we interviewed but extends to the total of 143 households tied into the network through transactions. The size of the nodes corresponds to their importance in the overall network (so-called betweenness centrality) and the thickness of lines, to the number of transactions (Freeman 1979). Reciprocal ties are black. Figure 1 supports the impressions gained through the above analyses. The visualization clearly shows a structured network. Half of the transactions (*N* = 254) are concentrated in the 30 most active social relationships. Transactions are thus not only reciprocal but also focus on specific ties.

### *Augu*—Reciprocity on Demand

The above analyses have shown that transactions are demanded and that all members of the community are entitled to demand. The social structure of food transactions that emerges is best described as reciprocal exchange, concentrating in specific ties. The results, best summarized as reciprocity on demand, perfectly match the people’s name for it: *augu*, asked to give (*au*) and to return (*gu*).

Even though one may ask anyone from the group for food, praxis concentrates on fewer and on reciprocal ties. As the analysis of the context in which transactions take place has revealed, the knowledge about whether someone possesses something at a given time, and its visibility, are essential both for demanding but also for evaluating the behavior of others. Most of the relatively small houses in Fransfontein are built of mud, dung, and wood. For most of the year, the heat makes it inconvenient to stay inside the houses during the day. Food is prepared outside. With a very open settlement structure and little vegetation, most neighbors are in sight. Hence, people easily recognize if someone in their neighborhood comes home with fresh loaf of bread, a bag of sugar, or even a piece of meat. As Bliege-Bird and Bird (1997:69) have pointed out, the costs of hiding and defending resources increase with proximity and foster transactions. At the same time, the costs of monitoring and sanctioning decrease with proximity and foster durable reciprocal relationships among neighbors. Other studies offer additional support for saliency of neighborhood ties (Allen-Arave et al. 2008; Gurven 2004; Hawkes et al. 2001; Widlok 2013; Ziker and Schnegg 2005).

**Fig. 2:**
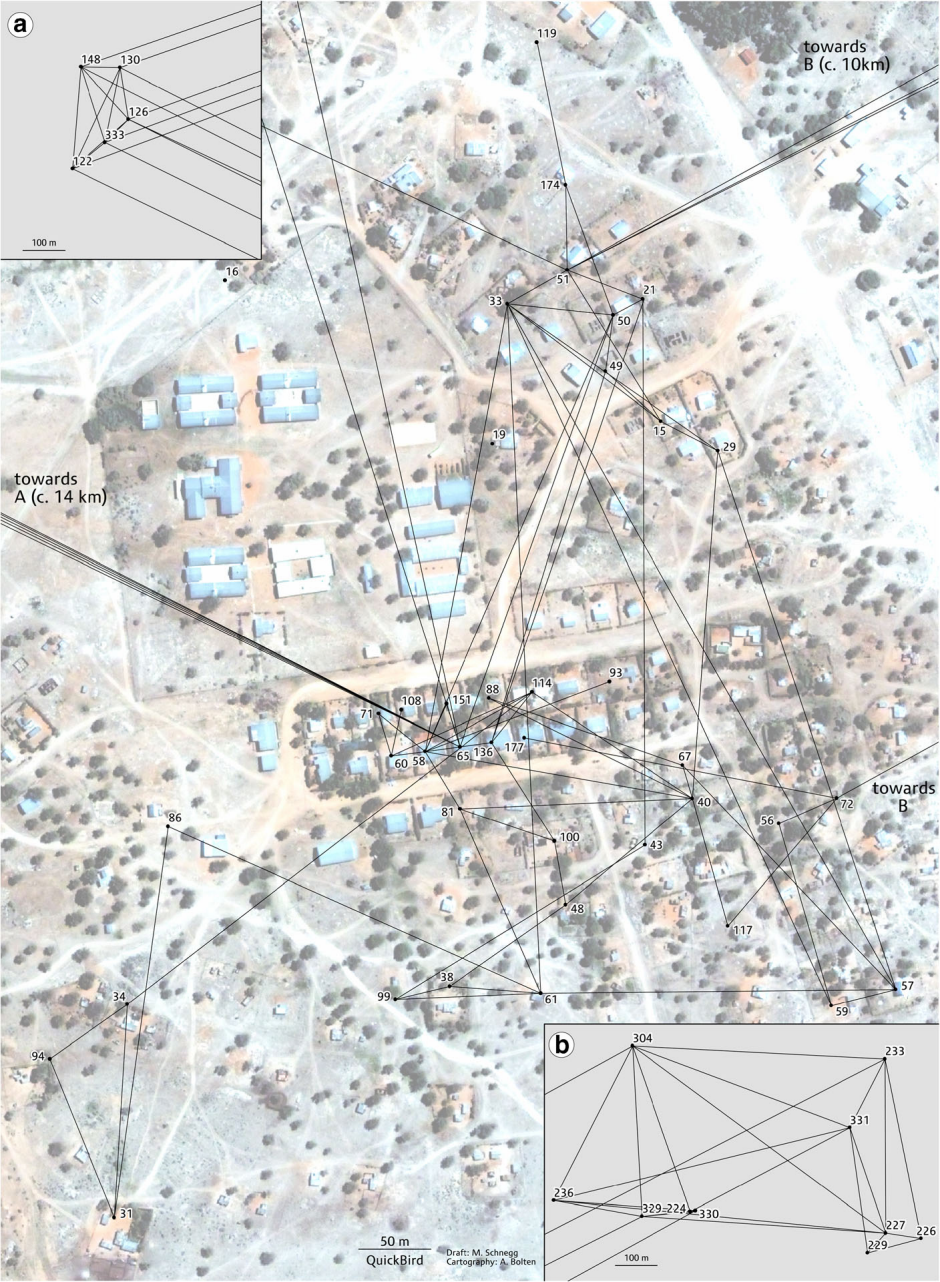
Geographic proximity and *augu* ties

This leads to the hypothesis that geographic proximity structures exchanges significantly, also reducing other costs involved in transacting food (e.g., transportation). To explore this dimension more explicitly, Fig. 2 takes a closer look at the interplay between space and transfers. Each household is placed on its geographical coordinates. Figure 2 hints that geographic proximity has an influence on the flow of goods, even within camps. Exchanges are not randomly distributed. We find clusters with a radius of few dozen meters around many households.

To put this visual interpretation to a stricter test, the two phenomena can be correlated. The Quadratic Assignment Procedure (QAP) has been developed to correlate two matrixes and to estimate the significance of the relationship (Hubert and Schulz 1976). To do so, I calculated a matrix of distances (in meters) between any two households. The resulting matrix contains 62 rows and columns, and each cell gives the distance between any two households. For the statistical test these distances were divided into three classes: <50 m, between 50 and 100 m, and >100 m. Those categories reflect immediate neighbors, neighbors within close walking distance, and those further away. QAP combines a regression analysis with a permutation test that accounts for the fact that entries in an interaction matrix are statistically not independent. The test provides those two statistics: the Pearson Product–moment correlation between the two variables and an estimated level of significance. The correlation between distance and food transactions is 0.198 and highly significant (0.000). The results of the statistical analysis thus support the visual interpretation of the data. Many *augu* transactions are in fact shaped by geographic proximity. However, even though the correlation is relatively strong, it does not account for all the variation in the data.

In the literature, kinship relatedness is often considered an alternative explanation (Ziker and Schnegg 2005). Unfortunately, my genealogical data are not sufficiently deep or accurate to test for kinship as an alternative model. However, from my ethnographic observations I suspect that close kinship is less significant for the relatively small transfers that constitute *augu*. Although residence patterns make kinship and geographical proximity overlap, I doubt that kinship relatedness offers much additional explanatory power. For ethnicity, a comparable test is possible. The results reveal that transactions are not restricted to the ethnic group (*r* = 0.03, ns) and confirm the ethnographic observation that practices do not differ between ethnic groups (Dawids et al. 2007).

Still, these findings leave room for further interpretations. A case study helps to account for some of this variation qualitatively. Olga, a female household head in her sixties, is a well-connected person who frequently exchanges with her neighbors. Consider two of the relationships in detail. On the one hand, Olga has a relation with Fiona. Like Olga, Fiona is a female household head and about Olga’s age. They both take care of a number of children and grandchildren and have regular access to pension payments. As the figure indicates, they exchange widely and on a regular basis. Flows go in both directions, the trust between the two is high, and the relationship is typical for the strong ties we have seen in Table 3 and Fig. 1. In contrast, the relationship with her other neighbor, Sarah, is much less extensive. When I talked to Olga about her *augu* relations during the interviews she explained that a few days before, her neighbor Sarah saw that she had slaughtered a goat and demanded a share of the meat. As Sarah put it: “Let me not only taste the smell but also the meat of it; give me some, give me some!” Olga remembers that she handed her a nice fat piece of the goat over the fence. During the following days, she ran out of sugar and sent one of her children to Sarah’s house to *au* a small quantity of it. At first, Sarah gave some sugar, but on the second day she refused, claiming that she had nothing herself. Olga was sure that this was a lie since she saw Sarah cooking and serving both the household and visitors. When Olga told me this, she explained that after having sent a child a few times, she stopped doing so. She felt that it was not worth getting angry and starting a quarrel when the solution was simple: just refuse to comply with future demands. The case highlights that Olga has an expectation that the demand would be reciprocated and thus that giving today indeed creates an expectation of entitlement to receiving tomorrow. If exchanges occur, they can become intensive, and many goods may be regularly transferred. However, if one party denies the demands of another, the potential for tension and conflict arises, and people are more likely to drop their demands and to give less in the future rather than risk conflict.

In sum, geographic proximity forms a context in which demands are easily expressed. Exchange relationships are built around certain dyads that develop trust and mutual dependency. This does not mean that all relationships will persist, but it does explain why some dyadic relations become intense and enduring bases for transaction relations.

## Conclusion

In the anthropological debate, two competing models have been used to conceptualize food transactions: sharing and exchange. Sharing transactions are interpreted as being formed through demands and spontaneous responses, whereas the notion of exchanges is connected with strategic networking. The debate originally focused on the ways meat is divided after a successful hunt of large game but has since been extended into other domains. Here I propose that the picture may be more blurred than this classification suggests.

To explore food transactions, I have offered a conceptual framework that differentiates two levels of the analysis: norms and practices. While the normative level is well understood from ethnographic interviews and observations, the social structures that evolve can be captured more precisely through quantitative data and network analysis. At first sight, the results of the analyses seem to contradict. The ethnographic data reveals clearly that people in Fransfontein demand basic food items regularly and as soon as excess food is in evidence. For those asked, it is difficult to refuse the requests. At the same time, network analysis based on actual transfers shows that the relationships involved in transactions are highly structured. They concentrate on reciprocal ties and fit the notion of exchange much better than that of sharing.

To explain how the two forms actually combine, two variables are crucial: geographic proximity and the internal dynamics of building and maintaining social ties. Proximity creates visibility and reduces information costs for those who demand. At the same time, proximity makes it costly not to give or to try to hide one’s gains. While people could in theory demand from anyone, in practice they often turn to their neighbors. However, even within the local context the strength of the relationships varies significantly. Specific interpersonal dynamics of trusting, depending, building, and maintaining relationships explain why some ties become (at least) temporarily strong and others not.

In behavior ecology, reciprocity is considered an ultimate explanation for food transfers (Trivers 1971). How reciprocity emerges requires proximate explanations and assumptions (Brosnan and de Waal 2002). One such explanation is strategic networking and keeping score. You give to those from whom you have received or expect to receive in the future. As simulations show, the rule results in highly reciprocal network structures (Schnegg 2006). The case presented here tells a different story. Although reciprocity results, it does not follow a tit-for-tat pattern between individuals. Instead, people can request from all members of the community, and the demand is typically met. However, within practical and structural constraints (the most important being geographic proximity) this heuristic can lead to reciprocity as well. Given the high frequency of interactions and the relatively low value of the goods transferred, it may even be more advantageous than the keeping-score approach. Under these circumstances, unconditional sharing, a norm that has been viewed as an alternative to exchange, can result in reciprocity—here, reciprocity on demand.^8^

The analysis presented here has a number of shortcomings. The level or reciprocity observed always depends on time and the transfers involved. A relationship that is nonreciprocal over a 10-day period may be reciprocated if something is given in return on the eleventh (or a subsequent) day. In addition, social interactions in small communities are multiplex and often balanced out through transfers in other domains (e.g., labor). However, both of these shortcomings would lead to underestimating the level of reciprocity, and consequently the level should rise if more relations and longer periods are considered, strengthening the evidence for demand exchange.

The fact that the amount of goods being transferred could not be quantified is a second shortcoming. This prevents me from asking whether those who give more also receive more in quantitative terms. In addition, a larger randomized sample would eliminate potential sampling errors and produce a more representative analysis. However, even in light of these limitations, the results are stable, as confirmed by the author’s long-term participation and ethnographic work in the region, and strong, as suggested by the significance of the quantitative measures.

Most of the food shared in Fransfontein is neither hunted nor gathered, and caution should guide comparisons with other such contexts. However, the distinction between norms and practices and the techniques and measures proposed offer a way to compare cases systematically. In contrast to our knowledge of how large game is divided, comparably little is known about how food that is bought in shops or produced from domestic practices is distributed. Wider and systematic comparisons of food transactions should reveal under which conditions sharing, exchanging, or blurred transactional forms are most likely to emerge and to prevail.

## Footnotes

1 Throughout the article, I use the terms “transactions” or “transfers” to include both sharing and exchanging.

2 The data are from a survey conducted with all households in the research area (*N* = 328) in 2004.

3 Unlike in other contributions to the debate (e.g., Gurven 2004; Nolin 2010; Ziker and Schnegg 2005), in this paper I do not provide a comprehensive test of different theoretical models (e.g., kin selection, reciprocal altruism, tolerated scrounging, costly signaling) that have been proposed to explain why people transfer food.

4 In 2004, 250 N$ could be exchanged for 35 USD. By 2014 the amount had risen to 600 N$.

5 Interview MS, 25.9.2014.

6 Interview MS, 25.9.2014.

7 Figure 1 was produced with the software package Pajek, http://pajek.imfm.si/.

8 I am very thankful to one of the anonymous reviewers for pointing this out to me so clearly.
